# Acute Fatty Liver of Pregnancy: A Diagnostic Challenge

**DOI:** 10.7759/cureus.36708

**Published:** 2023-03-26

**Authors:** Awanti Yemde, Anjali Kawathalkar, Anuja Bhalerao

**Affiliations:** 1 Obstetrics and Gynecology, NKP Salve Institute of Medical Sciences (IMS) and Research Center (RC) Nagpur, Nagpur, IND

**Keywords:** treatment and prognosis, differential diagnosis, acute fatty liver of pregnancy, liver diseases in pregnancy, swansea criteria, hellp syndrome

## Abstract

Acute fatty liver of pregnancy (AFLP) is a rare, catastrophic disease affecting women in the third trimester of pregnancy or the postpartum period. We report a case of 24-year-old G2A1 with GA of 35 weeks who presented with amenorrhea, nausea, fever, vomiting, headache and yellowness of eyes. The patient was diagnosed with severe preeclampsia with Intrauterine death (IUD) with hemolysis elevated liver enzymes low platelets (HELLP). Investigations showed hypoglycemia, low platelet count, and raised liver enzymes with an altered coagulation profile. The patient was kept in the Medicine Intensive care unit, induction was done with misoprostol, and she delivered an IUD baby. The condition of the patient deteriorated, and she developed pulmonary edema. Thus, she was intubated. Ultrasonography (USG) of the liver showed altered echotexture. The condition of the patient was then improved. AFLP requires a high index of suspicion for early diagnosis. Hypoglycemia in a pregnant woman without overt /gestational diabetes mellitus along with deranged liver panels and thrombocytopenia gives a clue in diagnosing AFLP. Timely diagnosis and intervention can reduce both maternal and fetal morbidity and mortality.

## Introduction

During the third trimester or the early postpartum era, a rare catastrophic complication called AFLP might occur [1]. The incidence of AFLP is 1:10,000 on average [2]. There is growing proof that acute fatty liver of pregnancy (AFLP) has a hereditary foundation and that the fetus's faulty mitochondrial fatty acid beta-oxidation is responsible [3,4]. Early detection of AFLP can be challenging since it mimics pre-eclampsia, viral hepatitis, and pregnant cholestasis. However, a thorough history and physical examination, along with laboratory and imaging tests, are frequently enough to make the diagnosis [5]. Better mother and fetal outcomes require fast delivery and intense supportive care [6].

## Case presentation

With an ultrasonogram showing an Intrauterine fetal death of 32 weeks, a 24-year-old G2A1 woman with a gestational age of 35+3 weeks presented with intermittent fever, nausea, vomiting, and headache for six days. She received an injection zofer, injection of Pantop, and tablet Dolo 650 mg SOS for the same at a private facility.

During the physical examination, the patient was awake, obedient, and aware of time, location, and people. Her blood pressure was 140/90, her heart rate was 124/min, and her breathing rate was 20/min. Her temperature was 99°F. She developed pedal edema, icterus, and urine albumin of +1. Heart, lungs, and respiratory system evaluations were all positive. There was no organomegaly, and the abdomen was soft. Fetal heart sounds were not audible, and the fundus was 32 weeks in height. The uterus was well relaxed at 32 weeks, and an intrauterine fetal demise was discovered via ultrasound.

Numerous laboratory tests were conducted. Hemoglobin was 13.7 g/dL, white blood count (WBC) was 15,450/cumm, and platelet count was 80,000/cumm, according to a complete blood count (CBC). Aspartate aminotransferase (AST) level was 213 U/L, alanine aminotransferase (ALT) level was 217 U/L, total bilirubin was 12.30 mg/dL, alkaline phosphatase was 534 U/L, serum albumin was 1.5 g/dL, and serum globulin was 4.8 g/dL, according to liver function test (LFT). Urea was measured at 61 mg/dL; serum creatinine was at 2.44 mg/dL, random blood glucose was at 68 mg/dL, and serum ammonia was at 73 mmol/L. There was 1077 mg% of serum lactate dehydrogenase (LDH), an International normalized ratio (INR) of 1.3, and a D-Dimer level of more than 10,000. HIV, HCV, and HBsAg serological tests all came out negative. Both the indicators for viral hepatitis and malaria were negative. A tentative diagnosis was made as hemolysis elevated liver enzymes low platelets (HELLP) syndrome. The patient received a loading dosage of Magnesium sulfate (MgSO_4_) in light of their state.

The patient was sent to the medical intensive care unit (MICU) for additional management. The patient's cervical os was 1 cm, her cervix was 30% effaced, and her station was at -2 per vaginally. Two doses of 50 mcg of misoprostol administered intravaginally, six hours apart, were used to induce labor in the patient. The patient's deep tendon reflexes (DTRs) were sluggish, she had decreased urine production with hematuria, and her breathing rate was 36 breaths per minute. At os - 3 cm, the membranes were artificially ruptured. The woman took ten units of vitamin K intravenously and gave birth to a 1.87 kg male stillborn baby. All precautions were taken to avoid PPH, Pitocin drip was started as soon as the stillborn was delivered. Placenta was removed by controlled cord traction. The patient has had intravenous albumin, injection piptaz, injection metronidazole, injection Meropenem 1 g, and tablet febuxostat.

**Figure 1:**
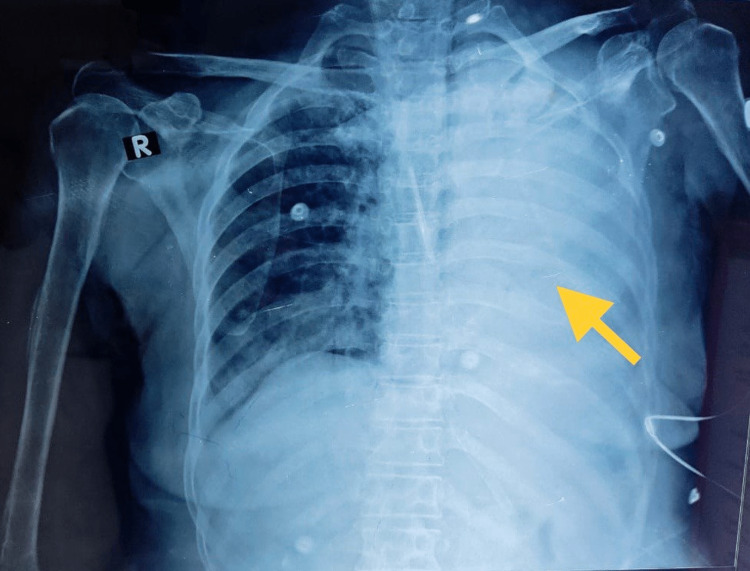
Chest x-ray showing left-side ground glass opacity indicating acute respiratory distress syndrome

Moderate ascites and a changed liver echo pattern were seen on abdominal ultrasound. As a result, the patient was diagnosed with AFLP. Hemoglobin began to decrease in the days that followed. Urea was rising from 61 to 99 mg/dL and creatinine went from 2.44 to 2.8 mg/dL. Aspartate and alanine aminotransferase levels increased in the liver function tests, and the INR was 1.58. Twelve hours after giving birth, the patient's condition began to deteriorate. Twelve units of fresh frozen plasma, seven units of platelets, and one unit of packed red cells (PRC) were transfused into her. The patient experienced acute kidney damage, disseminated intravascular coagulation (DIC), and pulmonary edema. For ten days, the patient was kept on regulated mechanical ventilation. Currently, a chest x-ray suggests acute respiratory distress syndrome (ARDS) (Figure 1). With time, her serum creatinine started to drop, her platelets increased, her hemoglobin improved, her bilirubin levels decreased, and her liver enzymes decreased. She slowly improved and was taken off the ventilator. On day 45, she was discharged.

## Discussion

The rare and dangerous pregnancy condition known as acute fatty liver (AFL) was initially identified by Stander and Cadden in 1934 and was once termed “acute yellow atrophy of the liver” [1]. Laboratory results and clinical symptoms are used to make the diagnosis of AFLP. Because AFLP exhibits unusual early symptoms such as nausea, vomiting, and abdominal discomfort, it is challenging to identify [7]. This instance emphasizes the significance of being watchful for pregnant women who exhibit jaundice.

Other possible diagnoses for jaundice during pregnancy include viral hepatitis, HELLP syndrome, cholelithiasis, and intrahepatic cholestasis of pregnancy (IHCP) [6]. These disorders' clinical symptoms and test results are ambiguous and nonspecific, making it challenging to distinguish between them [7]. Itching is the hallmark of intrahepatic cholestasis in pregnancy, and blood bilirubin levels seldom increase to 5 mg/dL. Any trimester can have cholelithiasis, which manifests with right upper quadrant pain and fever, and is diagnosed by ultrasound [8]. Any trimester of pregnancy can experience acute viral hepatitis symptoms, including fever, nausea, vomiting, tiredness, and jaundice. AST values are also significantly raised. In the third trimester, pre-eclampsia with liver involvement, HELLP syndrome, and AFLP manifest. HELLP syndrome occurs substantially more frequently than AFLP [9]. Laboratory abnormalities and symptoms of different liver and biliary diseases unique to pregnancy are shown in Table 1 [10].

**Table 1:** Laboratory abnormalities and symptoms of different liver and biliary diseases unique to pregnancy IHCP: Intrahepatic cholestasis of pregnancy, AFLP: Acute fatty liver of pregnancy, HELLP: Hemolysis elevated liver enzymes low platelets, TTP: Thrombotic thrombocytopenic purpura, HUS: Hemolytic uremic  syndrome, HTN: Hypertension, RUQ: Right upper quadrant, AST: Aspartate tranferase

	IHCP	AFLP	HELLP	Viral Hepatitis	TTP	HUS
Onset	late	late	late	variable	late	Late
Symptoms	Pruritus± jaundice	Nausea,Vomiting, HTN ,Preclampsia, RUQ pain	Preclampsia, RUQ pain	Jaundice , RUQ pain, fatigue	Neurological features+ fever +, purpura with bleeding	Neurological features±,renal dysfunction
AST	↑	↑↑	↑↑↑	↑↑↑↑	Normal	Normal
Creatinine	Normal	↑	↑	Normal	↑	↑
Platelets	Normal	↓↓	↓↓	↓	↓↓↓	↓↓
Hemolysis	Absent	↑↑↑	↑	Absent	↑↑↑↑	↑↑↑

Swansea criteria is used to diagnose AFLP, 6 or more features are suggestive of AFLP (Table 2) [10].

**Table 2:** Swansea criteria Wbc: Whole blood cells, AST: Aspartate transferase, ALT: Alanine transferase, AKI: Acute kidney injury, Cr: Creatinine

Clinical features	Laboratory features	Ultrasound features	Histologic features
Vomitting, Abdominal pain, Encephalopathy, Polydipsia /polyuria	Bilirubin >0.8mg/dL, Glucose <72mg/dL, Wbc>11,000/µL, AST or ALT >42U/L, AKI or Cr >1.7mg/dL, Ammonia >47µmol/L Coagulopathy or PT >14s, Urea >340umol/L	Ascites or echogenic liver	Microvesicular steatosis

Primigravida, male fetuses, past AFLP episodes, concurrent other pregnancy-related liver illnesses such as HELLP syndrome, and higher Body mass Index(BMI) are risk factors for AFLP [2]. According to the pathophysiology of AFLP, there is typically a physiological decrease in the oxidation of both long-chain and medium-chain fatty acids during pregnancy, which results in an elevated maternal fatty acid level in the mother. Due to foeatal dificiency in long chain 3 hydroxy acyl coezyme reduced b oxidation of fatty acids occurs and thus fatty acids starts builiding up of mothers [4]. Autosomal recessive inheritance is seen in AFLP. The G1528C gene homozygous mutation is connected to AFLP. Short-chain acyl Co A dehydrogenase (S-CHAD), medium-chain acyl Co A dehydrogenase (M-CHAD), carnitine palmitoyl transferase deficiency and mitochondrial trifunctional protein deficiency are further enzyme deficiencies linked to AFLP [4]. Accumulated fatty acids damage the liver and result in microvesicular fatty steatosis [2]. This hinders the formation of cholesterol, fibrinogen, and coagulation factors. Pancreatitis is one of the side effects of AFLP since fatty acids are also harmful to the pancreas. Reduced vasopressin levels, brought on by a poor liver function and low vasopressinase clearance, can result in central diabetic insipidus [7].

The rapid delivery of the fetus is the foundation of treatment. Comprehensive supportive care and therapy are required for complications like hypoglycemia, hepatic encephalopathy, disseminated intravascular coagulation (DIC), renal failure, hepatic failure, and acute respiratory distress syndrome (ARDS). In a tertiary care hospital with an Intensive care unit (ICU) setting, AFLP requires multidisciplinary teams, including intensivists, anesthesiologists, obstetricians, neonatologists, and blood banks. Before birth, the mother should be stabilized, which includes managing the airways and treating hypertension, hypoglycemia, electrolyte imbalances, and coagulation abnormalities. If coagulopathy is present, vaginal birth is recommended over a cesarean. Hemodynamic monitoring is crucial during the postpartum period due to the significant risk of bleeding brought on by coagulopathy. Fluids and blood products must be administered. It is essential to address problems, including pancreatitis and diabetic insipidus. Rarely is liver transplantation performed in AFLP cases due to liver failure. Transplantation of an orthotropic liver should be considered if an irreversible failure occurs.

## Conclusions

Uncommon, life-threatening disorders of the third trimester of pregnancy or the early postpartum period include AFLP. A thorough history, physical examination, laboratory findings, and USG are important to make the diagnosis. A hint to the diagnosis of AFLP is hypoglycemia in a pregnant woman without overt or GDM along with deranged LFT, or thrombocytopenia. The cornerstones of treatment for AFLP are prompt delivery of the foetus and rigorous supportive care.
